# Working in partnership to reduce re-offending and improve prison leavers’ lives: a process evaluation of a prison leaver pilot project

**DOI:** 10.1080/10509674.2024.2406748

**Published:** 2024-10-09

**Authors:** Hayley J. Lowther-Payne, Ella Whitcomb-Khan, Fiona Ward, Iliana Makri, Nicola Gaskins, Joanna Goldthorpe, Paula Wheeler

**Affiliations:** a Applied Health Research Hub, University of Central Lancashire (UCLan), Preston, Lancashire, UK; b School of Sport and Exercise Sciences, Liverpool John Moores University (LJMU), Liverpool, UK; c Division of Health Research, Lancaster University, Lancaster, Lancashire, UK; d Active Lancashire, Leyland, Lancashire, UK

**Keywords:** prison, sport, peer mentoring, qualitative; re-offending; partnerships

## Abstract

Prison leavers encounter significant barriers to successfully re-integrating into the community, which can lead to re-offending. Complex interventions which are multi-faceted and involve successful partnerships are needed to meet the distinct health and social needs of this population group. For this study, we conducted a process evaluation of a pilot project, which aimed to offer holistic support to prison leavers through a combination of peer mentoring, sport and physical activity, and signposting, delivered in a community setting. Semi-structured interviews and focus groups were conducted with individuals (n = 14) involved in the delivery and the management of the project to understand how it had been implemented and what factors had influenced the delivery and partnerships involved. Factors that influenced project delivery included safeguarding and risk assessment concerns, lived experience of peer mentors, accessibility of the intervention, and the role of sport and physical activity as a vehicle for community re-integration. Partnership working was influenced by effective information sharing, organizational commitment, building relationships and professional networks, and regular communication between organizations. An intervention involving peer mentoring, sport and physical activity, and signposting, supported by close partnership working, was viewed as a promising approach to support the community re-integration of prison leavers.

## Background

Individuals in contact with the criminal justice system experience significant health inequalities, including a higher prevalence of complex health and social needs, low levels of help-seeking behavior, and an increased risk of premature death (Revolving Doors Act, 2017). Prison leavers specifically encounter many barriers to successful community re-integration such as a lack of continuity of care, stigma associated with being in prison, social isolation, lack of educational attainment, unemployment, and housing issues (Buck et al., 2021; Tarpey & Friend, 2016), which perpetuate health inequalities (Burgess-Allen et al., 2006). The need for interventions to address the barriers faced by prison leavers and provide support which meet the needs of this population group is nationally recognized (National Offender Management Service, 2004; Revolving Doors Act, 2017).

Existing interventions within the UK criminal justice system have predominantly been delivered to individuals within the prison setting prior to their release. For example, mentoring programmes that involve support being delivered by *peers* who have lived experience of the criminal justice system (Bagnall et al., 2015; Fletcher & Batty, 2012; South et al., 2014;), and sport and physical activity programmes that enable prisoners to participate in activities aimed at improving physical and mental health during their incarceration (Ministry of Justice, 2018). These interventions are limited to their application only within the prison setting and often adopt a single approach (e.g., peer mentoring or sport and physical activity) to support those in contact with the criminal justice system. Given the distinct set of complex health and social needs of prison leavers, multi-faceted interventions that take a holistic approach and involve a range of organizations are needed to address the barriers individuals face upon their release from prison. However, *how* these interventions are delivered to support prison leavers in the community and *what works* in terms of their implementation is rarely examined. Whilst evaluating the effectiveness of an intervention is useful to ascertain whether it works, conducting a process evaluation of an intervention enables assessment of its implementation (how it works), clarification of the causal mechanisms (why it works), and identification of contextual factors that influence its delivery (Moore et al., 2015; Skivington et al., 2021).

Despite the recognized importance of continued support on release from prison (Tarpey & Friend, 2016), few examples of community-based interventions for prison leavers have been described previously (Hosking & Rico, 2018; Hough, 2016; Revolving Doors, 2022; Wadia & Parkinson, 2015), particularly those which involve utilizing a holistic approach. Across these examples however, it is clear that third sector organizations are considered to play a fundamental role in the delivery of community re-integration interventions for prison leavers through their position within the community and their partnerships with other organizations (Clinks, 2018; Mills et al., 2019). Effective multi-agency working and building partnerships are frequently noted as critical elements of success for the delivery of these interventions, but there is little reported about the factors which influence how these partner organizations work together. This study aimed to address a gap in the literature by conducting a process evaluation of a pilot project, which was multi-faceted in nature and offered holistic support to prison leavers through a combination of peer mentoring, sport and physical activity, and signposting, delivered in a community setting.

### The pilot project

The pilot project evaluated in this study was a collaborative effort between a third-sector organization, the regional probation service, and seven local community football trusts. The delivery of the project was focused across eight areas of one county in North West England with particularly high re-offending rates. The project’s aim was to support prison leavers who were under probation supervision to successfully re-integrate into the community, improve their health and wellbeing, and reduce re-offending. Individuals who took part in the pilot project were referred from the probation service or other similar organizations subcontracted by the Ministry of Justice. The project team operated within the community and contacted the referred individuals to see whether they were interested in taking part. There were no eligibility criteria for participation, other than a need to want to improve their lives. The project did not differentiate by offense, and although referral of high-risk individuals was rare, the approach to manage risk was tailored to each individual.

**Figure 1.**
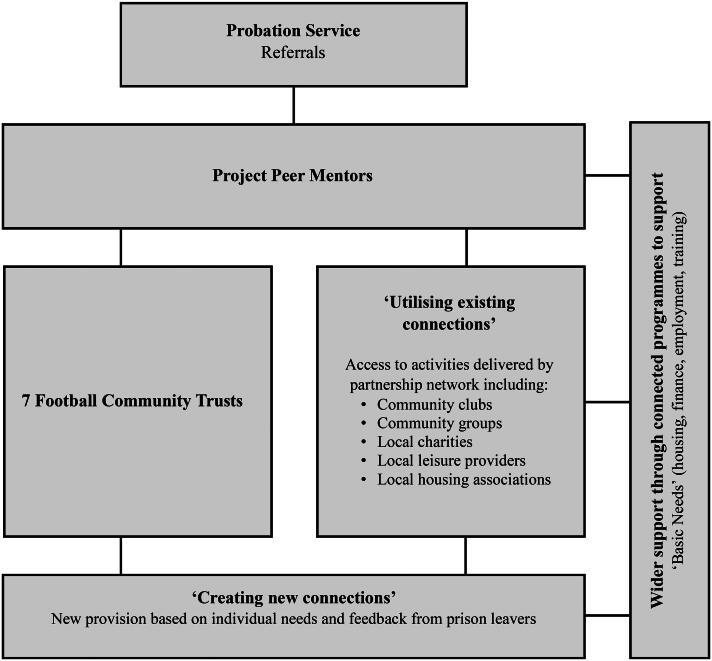
The pilot project delivery model.

Each participant was matched with a peer mentor, who they had regular one-to-one meetings with to discuss their needs and create an action plan personalized to the individual and the changes they wanted to make in their lives. The participant and their peer mentor, who had lived experience of the criminal justice system, worked in partnership to agree an offer of support, which included activities provided by community football trusts, support offered by other organizations in the partnership, and signposting to a wider range of local service provision. Community football trusts provided advice on health and nutrition and access to sport and physical activity sessions allowing prison leavers to socialize and expand their social networks in a safe environment. The third sector organization which acted as lead for the pilot project was a community-based, nonprofit organization working locally to support community engagement in sport and physical activity. This organization provided access to activities delivered by services in the local area such as community clubs and groups to encourage reintegration into the community. Individuals were also signposted by their peer mentors to a wider range of service provision through established partnerships to address their basic needs, such as support for housing, finances, mental health, substance misuse, employment, and training opportunities. Individuals accessed services which were most relevant to them in agreement with their peer mentor, therefore not all participants accessed all of the types of support offered. Based on the needs and feedback from prison leavers, new provision was sought where existing provision was insufficient. The pilot project delivery model is presented in Figure 1. Where participants were recalled to prison or had re-offended and were sentenced again, the project team attempted to maintain contact with the individual so that they could be met on release from prison and supported to re-start the project.

### Research questions

The aim of this study was to apply a process evaluation framework to explore the experiences and perspectives of those involved in managing and delivering the pilot project described above to answer the following research questions:

1. How has the project been delivered, and how have partner organizations worked together?
2. What factors have influenced the delivery of the project and the partnerships involved?
3. To what extent is the project sustainable and could be easily replicated in other areas?

## Methods

### Study design

This was a qualitative, semi-structured interview and focus group study conducted between June and October 2021. The methods used and analysis conducted in this study are reported in accordance with the Consolidated Criteria for Reporting Qualitative Research (COREQ) (Tong et al., 2007). A checklist is presented in Appendix 1.

### Theoretical framework

The project under evaluation involved a range of organizations working in partnership to deliver a multi-faceted program of support, ultimately aimed at individuals leaving prison to overcome barriers to community re-­integration, improve health and wellbeing, and reduce re-offending rates. Therefore, understanding the factors that have influenced the delivery of this complex project and the partnerships involved is important. The Medical Research Council (MRC) process evaluation guidance offers a theoretical framework by which the implementation of complex interventions can be subjected to evaluation, rather than just their effectiveness (Moore et al., 2015; Skivington et al., 2021). The framework alludes to *implementation* as the structures, resources, and processes through which delivery is achieved, *mechanisms of impact* as how the intervention evokes change in outcomes, and *context* as how external factors influence the delivery of the intervention. As preliminary data had already been collected and analyzed to measure the outcomes of the project and reported separately, the focus of this evaluation was to explore factors associated with its implementation.

### Ethics and governance

Ethical approval for this study was obtained in August 2021 from Lancaster University’s Faculty of Health and Medicine Research Ethics Committee (FHMREC20158). A research steering group, made up of researchers, representatives from the lead organization, and members of the public with lived experience of the criminal justice system, was convened for this study. The group met regularly to advise on practical arrangements (e.g., participant recruitment), provide information on the wider context (e.g., current policy, practice), and offer insight into the findings of the study as they emerged.

### Participant recruitment

A purposive sample was identified through the lead organization of the project. Representatives from partner organizations involved in managing and delivering the project were formally invited to take part in the study via email sent by one researcher (EW). Organizations invited to take part included a third sector organization (leading the project), a community rehabilitation company, seven community football trusts, the probation service, a local prison, and a local housing association. Those invited to take part were sent a participant information sheet via email and were asked to complete a consent form prior to the date of the interview or focus group. A total of twenty individuals were invited to take part in the study; of those, fourteen individuals replied and took part in either a one-to-one interview or a focus group.

### Data collection

Nine semi-structured interviews and two focus groups were conducted by researchers (EW, FW, & HL) with representatives from partner organizations involved in managing and delivering the project; one third sector organization (*n* = 6), four community football trusts (*n* = 5), the probation service (*n* = 2), and a local prison (*n* = 1). Due to unsuccessful recruitment attempts and the short timescale for study completion, researchers were unable to interview representatives from the community rehabilitation company and the local housing association. Overall, six individuals in strategic roles (e.g., directors, managers) were interviewed, and eight individuals in operational roles (e.g., peer mentors, support workers) were interviewed (*n* = 3) or took part in small focus groups (*n* = 5). One-to-one interviews were predominantly conducted with individuals in strategic roles as their perspectives were unique to their role and for pragmatic reasons. Focus groups were conducted with individuals in similar delivery roles as this was suggested by the steering group to help with prompting discussion amongst mentors and reduced the time and resources required for data collection. Researchers (EW, FW, & HL) had previous experience in conducting interviews and focus groups and held research positions with their institutions.

Participants were asked to reflect on their experiences of the project and provide their perspectives on how it was implemented, the value of the partnership involved, the barriers and facilitators to project delivery and partnership working, and the project’s sustainability. An interview or focus group schedule with a series of questions was used by the researcher to facilitate the discussion and maintain consistency, and additional prompts were used where necessary. The schedules were developed by researchers, reviewed by the steering group, and edited based on feedback provided by the group. Copies of the schedule are available on request from the corresponding author. Interviews and focus groups, conducted remotely using Microsoft Teams or over the telephone, were audio recorded and transcribed verbatim by an independent contractor. Field notes were also taken by the researcher during the interviews and focus groups to accompany the transcripts. The average length of the interviews and focus groups were 47 minutes and 73 minutes, respectively.

### Data analysis

Transcripts from the interviews and focus groups were anonymized and imported into NVivo 12 (QSR International Pty Ltd. NVivo (Version 12), 2018) for analysis. Two researchers (EW & FW) initially read and re-read the transcripts to immerse themselves in the data. A deductive thematic analysis approach, as outlined by Clarke et al., (2015), was used to inform the coding of the data and the development of themes. Existing concepts in the MRC process evaluation framework (Moore et al., 2015; Skivington et al., 2021) of *Implementation* (how was the intervention implemented?), *Context* (what was the influence of contextual factors?), and *Mechanisms* (how does the intervention impact on outcomes?) were adopted as overarching themes to deductively inform the analysis and interpretation as this was identified to be most suitable for the research questions.

Two researchers (EW & FW) coded the data in NVivo 12 (QSR International Pty Ltd. NVivo (Version 12), 2018). Codes were synthesized into categories, and sub-themes were formed under the concepts of the MRC process evaluation framework. An inductive approach was adopted to reflect on any data that did not fit into these concepts. EW and FW met during the coding process to review emerging themes, compare the coding, and check for agreement. Field notes were referred to where necessary to expand on the interpretation of the transcripts. The emerging themes were shared and discussed with the steering group during the data analysis to ensure that the data had been interpreted appropriately and provided an opportunity for feedback.

## Results

This section is organized according to whether findings are associated with the delivery of the project or to the partnership working involved, as per the research questions. Key themes are summarized accordingly in Tables 1 and Table 2, and described in the sections below with examples of participant quotations.

**Table 1.** Summary of themes related to project delivery, according to MRC framework concepts (Moore et al., 2015).

MRC process framework concept	Theme	Summary of key points
**Implementation**internal factors affecting delivery	Safeguarding and risk assessment	Views of risk differed between community football trusts and other partner organizations Data management system used in the project was felt to provide insufficient detail to assess level of risk
	Clarity on community football trust offering	Some partner organizations were unclear about community football trust services on offer and what was required of the prison leavers they were referring Some partner organizations expected that services on offer from community football trust to be exclusive for referred prison leavers
**Context**external factors affecting delivery	COVID-19 pandemic	Services were reduced due to the pandemic restrictions, and this impacted on the delivery of community football trust services Continuation of peer mentoring services during the pandemic was valued
	Sustainability	Funding was viewed as short-term and inadequate
**Mechanisms of impact**factors contributing to successful delivery	Individual characteristics	Lived experience of peer mentors Enthusiasm and integrity of staff
	Relationship building with prison leavers	Perseverance of peer mentors Adopting a person-centred approach
	Accessibility and sustained delivery	Convenient location of services (e.g., close to public transport links) Inclusive approaches to referrals
	Community football trust involvement	Credibility and appeal of well-known Football Clubs involved Quality of the facilities offered
	Physical activity as a vehicle for change	Appropriate use of free time Learning new skills

**Table 2.** Summary of themes related to the partnership working, according to MRC framework concepts (Moore et al., 2015).

MRC process framework concept	Theme	Summary of key points
**Implementation**internal factors affecting delivery	Planning and information sharing	Clear, documented agreements of ways of working from the outset of the project (e.g., information sharing, referral procedures, risk assessments, clarity around roles, service offers)
	A long-term shared approach	All partnership organizations involved in strategic planning and decision making, and kept up to date on project developments All partnership organizations share information on funding opportunities and collaborate on applications for funding rather than compete
**Mechanisms of impact**factors contributing to successful partnerships	Building professional networks	Building good professional networks led to successful referrals and signposting to services
	Communication	Good practice in terms of communication (e.g., regular meetings supplemented with ad-hoc calls for specific advice and information sharing when necessary)
	Relevant skill mix	Partnership organizations respected as having expertise relevant to their own field Partnership organizations had complementary skills to deliver the project

### Project Delivery

#### Implementation

Safeguarding and risk assessment was a somewhat contentious issue between organizations and was seen to largely rely on the level of experience of working with prison leavers. Safeguarding was a particular concern for community football trusts, who were more inexperienced in working with prison leavers, and so were often viewed as more risk-adverse than other organizations. This was thought to have influenced the implementation of the project as referrals to take part in activities were sometimes not accepted by organizations and sessions in shared spaces with other population groups, such as young people or vulnerable adults, were changed due to concerns about safeguarding and assessing the level of risk. There was a perception that these actions could lead to stigmatization and disengagement of prison leavers, which would have ultimately influenced the way in which the project was delivered. A data management system was implemented as part of the project to share information between organizations and was considered as a potential solution to addressing concerns about safeguarding and risk assessment. Whilst sharing information about prison leavers and their offenses may have supported improved risk assessment and organizations to make suitable adjustments to their activities, the system was viewed as unpopular with some participants. There was a tension between protecting the confidentiality of prison leavers and providing sufficient detail to determine risk and inform safeguarding measures. As a result, there were missed opportunities for some individuals taking part in the project to benefit from activities offered by the community football trusts.

*“a lot of the referrals were bounced back because they (community football trusts) were just quite hesitant, they’d never done this nature of work before”* [third sector organisation]

*“I’d rather see that you know clearly […] it’s not to say that we can’t work with them people ‘cos they’re too high risk or whatever, it’s more just that we’re informed, and we can put things in place” [community football trust]*

There was some uncertainty about the activities that were being offered by community football trusts. Peer mentors and referrers from the probation service reported finding it difficult to communicate the activities the community football trusts were making available to prison leavers as a result of this lack of clarity. This led prison leavers to often assume that activities would only be related to football or would present at sessions unsure of what they were getting involved in. Peer mentors suggested that community football trusts should be clearer about the activities on offer besides football, such as general sport sessions and skills development, and that any activities made available to prison leavers should be targeted and be provided exclusively to them. Participants reported that the sessions were open to other groups, leaving peer mentors and referrers from the probation services unsure whether the activities they were signposting to were exclusive to, and suitable for, prison leavers and if it still offered the same peer support element.

*“I didn’t really understand what was on offer […] I think they were just turning up and it they weren’t really sure what they were getting involved in”* [third sector organisation]

*“having more participants who were not criminal justice system referrals – it has diluted the services. It has impacted on a key component which was the support participants got from each other […] they all sort of that peer-to-peer support and it was really working”* [community football trust]

#### Context

The COVID-19 pandemic and associated measures to contain the spread of virus resulted in the temporary closure or restricted offering of services, and so support from some services as part of the project was limited during these time periods. Face-to-face peer mentoring provided by the third sector organization did not stop during the COVID-19 pandemic. Due to barriers associated with prison leavers accessing online support and the perception that concerns may not be identified during remote contact with peer mentors, the continuation of face-to-face support was valued by referrers. The short-term nature and inadequate provision of funding available for the project was thought to have impacted on delivery. As such, the project was perceived as unsustainable in its current funding status by the majority of participants, with more investment desired to maintain the relationships that had been built.

*“the probation service couldn’t go out face to face to see people, we could […] they were ringing people up that were presenting really well on the phone, but I was seeing them face to face, reporting back to probation saying no they are not at all well”* [third sector organisation]

*“it’s been difficult in that the funding just doesn’t go anywhere near to what we’ve been trying to deliver”* [community football trust]

*“we’ve run out of funding now haven’t we, we haven’t got funding now and it’s a shame because all of them relationships are built”* [third sector organisation]

#### Mechanisms of impact

Individuals with lived experience of the criminal justice system acting as ‘peer mentors’ were reported as having a greater appreciation of prison leavers’ needs and experience, such as stigmatization, negative public attitudes, and challenges in accessing support. This form of peer mentorship was viewed as essential for initiating and sustaining engagement with prison leavers. Enthusiasm, a passion for the role, a caring nature, and the ability to demonstrate these qualities to engage with prison leavers were seen as useful characteristics for staff delivering the project. Staff were felt to be respected by prison leavers when they showed integrity in their approach to the work, and this reliable and consistent provision of peer support facilitated better relationships with prison leavers. Knowledge of, and links within, the local community were also valued by staff with lived experience, as it supported their ability to signpost prison leavers to the most appropriate service. In addition to personal attributes, it was important for peer mentors to receive appropriate training to enable them to apply their lived experience in a way that was useful to prison leavers.

*“they (peer mentors) can talk about real life experiences, and able to say, I’ve been where you’ve been and now I’m here and this is what I did to get here, and I feel like that’s so powerful”* [probation service]

*“he’s (peer mentor) open, he’s honest, and he gets involved, and he cares, and his enthusiasm is just unbelievable, and I think you need that, if you’ve not got that you kind of I think you’re fighting a losing battle”* [community football trust]

Peer mentors’ willingness to persist in working with prison leavers who would not initially interact with the project was viewed as key to engagement, building relationships, and encouraging successful outcomes. This was particularly valued by the probation service who had limited resources to sustain engagement work with prison leavers. A person-centred approach was reported by participants as crucial to building sustained relationships with prison leavers. Being nonjudgmental, assessing their needs, and developing a tailored package of support to respond to those needs was thought to demonstrate that staff genuinely cared about prison leavers and facilitated prison leavers being more likely to follow staff recommendations.

*“they’re (peer mentors) good at trying to support that person where they need to get to, and they don’t give up […] really important with the people we work with because it might take six times before somebody goes […] I’ve had enough of this bouncing in and out of prison”* [probation service]

It was important for project delivery that sessions were held in a convenient location, well connected with public transport, and ideally in places familiar to prison leavers (e.g., town centres). In addition to physical accessibility, the easy referral process was thought to facilitate prison leavers’ access to services and increased the likelihood of further referrals. A policy of not turning anyone away meant that the project could accommodate more challenging prison leavers and referrers felt that they could trust that the individuals they referred to the project, even if higher risk, would be supported.

*“what makes it successful is building trust with each client (prison leaver) and being proactive and what made us successful […] we could always find somewhere for them to go […] we wouldn’t give up”* [third sector organisation]

*“we put on a weekly session in the centre of (town) so that it was easy for them to get to”* [community football trust]

Community football trust involvement was viewed as a key component of the project. Participants reported that initially the association with a well-known football club acted as a ‘hook’ for prison leavers to engage with the project. It was also viewed as an important way of getting buy-in from the probation service. Community football trusts offered desirable resources, facilities, and opportunities well-received by prison leavers, such as volunteering and tickets to matches.

*“it was the pull of the badge”* [probation service]

*“there’s no denying that people hear a football club are involved and it’s like I want to be involved with them so I think you know it’s a really good sort of way to get people engaged”* [community football trust]

*“it was the badge and they had the facilities and if the right person went along to the football club, there was endless amounts of support they could get”* [third sector organisation]

Physical activity was viewed as integral to the overall programme of support and was thought to provide prison leavers with a productive and beneficial way of spending their free time and reduce chances of engaging in criminal activity. Participants reported that participation in physical activity provided prison leavers with a myriad of opportunities to improve their wellbeing and reduce chances of re-offending, such as access to peer and social support, developing a sense of belonging to a wider community, trying out new activities, and learning new skills.

*“we got people active, we got them doing something, filling their time productively, you know with positive stuff”* [community football trust]

*“if people are attending sessions, they genuinely feel a part of something, so it’s about that sense of belonging”* [community football trust]

### Partnership working

#### Implementation

Effective information sharing between organizations early in the project was viewed as vital to promote clarity around the activities on offer and what the expectations were for prison leavers. Formalizing a joint understanding between organizations with clear, documented agreements on ways of working from the outset of the project was noted as a way of addressing this lack of clarity. For example, developing guidance on referral procedures, risk assessments, defining roles and service offers, and information sharing processes before the project commenced. The absence of this early project planning was thought to have led to some prison leavers being excluded or experiencing delays in accessing the support available, and hampered service delivery such as difficulties for peer mentors accessing prisons to initiate support. Organizations involved in strategic planning and decision-making being committed to partnership working in the long-term was viewed as important, and without this, the delivery of the project would be ultimately less successful. Competition between organizations for a finite supply of funding was noted as a barrier to partnership working.

*“we could really do with a workshop somewhere along the line just management going into probation offices and actually explaining what our service does”* [third sector organisation]

*“the hard thing was the lack of clarity from them really at the beginning about what, you know we were a bit new to it and they were kind of telling us what they needed but they didn’t have many ideas of what they wanted it to look like”* [third sector organisation]

*“big breakdown early on there was a lot of safeguarding issues to come in and some of the (partner organisations) just couldn’t […] it’s not just we’re picking out people who’ve done minor assaults, you know they are an absolute variety of people that are coming in”* [third sector organisation]

*“I’m saying to them we need to apply for the (funding), three of them went and applied on their own before we realised, can you just retract that because we’re trying to all go in together”* [third sector organisation]

#### Mechanisms of impact

When individuals working in the partner organizations had been in their role for some time, successful relationships had developed over time which facilitated more referrals to the project and timely access to support. Professional networks, which developed between organizations, enabled signposting to resources to support sustainability such as identifying new funding opportunities and working together on applications. These professional networks also supported organizations to adopt a more integrated approach to working with individual prison leavers, meaning that a package of support could be developed and delivered between partner organizations in a way that was tailored to their needs.

*“it’s the signposting that we can do […] the networks we’ve built up, the partners that we can work with and the trust we’ve got with those people”* [third sector organisation]

*“working in partnership just creates that better wrap around service which is giving that one person a better chance in life”* [third sector organisation]

All participants reported communication as essential for partnership working. Relationships between mentors and community football trust staff were supported by ad-hoc opportunities to communicate (e.g., telephone), and regular meetings to share good practice and discuss individual cases. Regular meetings facilitated information sharing and built trust between partner organizations. For example, peer mentors would accept referrals with less information as they trusted that probation service staff would make appropriate judgements on the suitability of those being referred, and the probation service increased the number of referrals they sent through as they felt more confident in the effectiveness and suitability of the project.

*“they (partner organisations) have a meeting once a month and discuss good practice or just have a general chit-chat about how it’s working in each area and how each other’s doing, so that’s worked really well”* [third sector organisation]

*“we do hold […] bimonthly steering group meetings where we’ll discuss sort of the operation […] and what was going well, what wasn’t going well”* [community football trust]

Partnership working was viewed as vital to coordinate the key components of the project (e.g., peer mentoring, participation in sport and physical activity, signposting), and each organization was thought to bring their own expertise to execute this complex project. Participants reported that this mix of skills and knowledge in the partnership improved safeguarding and enabled prison leavers with more complex needs to access services. Community football trusts had a unique role in the partnership as the provision of attractive resources and facilities, along with the reputation of being associated with the football clubs, were not only appealing to prison leavers but also to probation service staff.

*“with probation we’re very structured in what we’ve got on offer, so we had got (a housing association) providing accommodation, we’ve got the (a third sector organisation) providing support for women […] (the project) really filled the gaps for me on a personal level in relation to parts of offending behaviour we would look at around things like lifestyle and associates […] community re-integration”* [probation service]

## Discussion

This study applied the MRC process evaluation framework to explore the perspectives of those involved in managing and delivering a pilot project aimed at supporting prison leavers re-integrate into the community through peer mentoring, sport and physical activity, and signposting, with close partnership working. The study findings suggest that those involved in delivering and managing this complex intervention viewed it as a promising approach to support prison leavers to re-integrate into community and highlighted a number of factors that should be considered in the future implementation of this type of intervention. The findings and implications of this process evaluation are discussed in the context of existing literature in the sections below.

### Key findings in the context of existing literature

A number of factors were attributed by study participants to how the intervention contributed to impact. Mentoring from those with lived experience of the criminal justice system was viewed as integral to successful project delivery as it supported initiating and sustaining engagement with prison leavers. Research has highlighted that whilst peer mentoring can be a positive experience for mentors by providing a safe space where they can be themselves, it can also involve ongoing stigma, emotional burden, and career limitations for peer mentors based on their past (Buck, 2021; Buck et al., 2021; Nixon, 2020). Peer mentors interviewed in this study reported their involvement as a positive experience and their ability to relate to and engage with prison leavers was valued, particularly by those from the probation service. Training for peer mentors to use their lived experience appropriately was viewed as important and recommended. Buck, (2021) reported that the training, support, and supervision of peer mentors not only supports the delivery of peer mentoring but safeguards peer mentors and their wellbeing.

Opportunities for participation in physical activity were considered as an integral part of the project in order to support prison leavers to improve their wellbeing and reduce chances of re-offending. However, there were challenges, particularly on the clarity of what was being offered and whether it was exclusive to prison leavers, that needed to be addressed to support the implementation of these activities. A special interest group convened in 2016 highlighted factors which were thought to be essential for effective through-the-gate mentoring (Reducing Re-offending Third Sector Advisory Group, 2016). If the description of the mentoring and services being offered to prison leavers was unclear, it was reported that this could affect the quality and appropriateness of the delivery and as such may affect the benefits that could be achieved through mentoring prison leavers.

Unlike many services, this project did not close during the COVID-19 pandemic and continued delivering face-to-face support in line with regulations, which was particularly valued by those from the probation service. A significant increase in the early release for low-risk prisoners was seen at the start of the COVID-19 pandemic (UK Parliament, 2020). Had the project temporarily closed or altered its delivery methods, interviewees felt that many of these prison leavers would have faced barriers to accessing services without the continuity the project offered. People in contact with criminal justice system experience stigma, social exclusion, and poverty (Tyler & Brockmann, 2017), as such may not have been able to access the service if delivered remotely like many other services. The accessibility of this intervention as a whole was recognized as key to facilitating implementation in this study. Buck (2021) noted, similarly to the findings of this study, that an insecure funding environment has a significant impact of the delivery of interventions such as the one presented here and could result in the loss of services and partnerships, which has a detrimental impact not only on prison leavers but the peer mentors delivering the intervention.

In the Ministry of Justice (2013) report on “Transforming Rehabilitation: A Strategy for Reform”, partnership working was noted as an integral part of any programme to support the rehabilitation of people in contact with the criminal justice system, suggesting that a joined-up approach was required in this context (Ministry of Justice, 2013). Consistent with this study’s findings, Lennox et al. (2021) found that communication and information sharing influenced partnership working in their exploration of developing a complex intervention to support prison leavers with common mental health problems (Lennox et al., 2021). The role of third sector organizations has become increasingly important in engaging prison leavers and addressing the social determinants of health (Buck et al., 2021). However, without establishing effective partnership working with publicly funded services, such as probation, and securing adequate funding, the sustainability of programmes to address the needs of those leaving prison is unlikely. Safeguarding and risk assessment concerns are recognized barriers to working with prison leavers across the existing literature (Buck et al., 2021; Lennox et al., 2021). This study found that partnerships were affected by differing views and solutions to assessing risk which ultimately had a negative impact on the delivery of the project.

### Implications for practice

A holistic approach to support prison leavers, which involves peer mentoring, sport and physical activity, and signposting, was valued by those involved in delivering and managing the project. This evaluation has highlighted the importance of partnership working and the benefits of communication and information sharing amongst different organizations to deliver a complex intervention to meet the distinct health and social care needs of a population group experiencing significant health inequalities. Understanding factors that influence the delivery of this intervention and the partnership working involved, by applying the MRC process evaluation framework, has provided an understanding of how to optimize its future implementability. Enhancing mechanisms through which impact is secured (e.g., lived experience, participation in sport and physical activity) and addressing the barriers to the project’s implementation will undoubtably improve the deliverability of the intervention. Many of these factors could also be applied to enhance the implementation of similar interventions that aim to support prison leavers in re-integrating into the community.

### Implications for research

The MRC process evaluation framework (Moore et al., 2015) was successfully applied in this study to understand how a complex intervention to support prison leavers has been implemented and has raised important considerations for future research in the area. Relying on outcome measures, such as re-offending rates or the health and wellbeing of prison leavers, produces a snapshot of how individuals respond to an intervention and does not deliver a more in-depth understanding of the complexity of how a multi-faceted intervention might work. This study has provided insight into how the lived experience of peer mentors and the appeal of football clubs might act as mechanisms for impact, whilst considering contextual factors such as accessibility of the intervention and effective communication between organizations as key to implementation. This paper reports an evaluation of a small-scale pilot project conducted in one county in the North West of England, with prison leavers who were predominantly male. Whilst many of the findings may be generalizable, it is acknowledged that further research is needed to understand how the implementation of such an intervention may be influenced by different contexts, such as a different geographical area or population group. A large-scale implementation and evaluation of the intervention could be used to assess how the pilot project could be rolled out and scaled up in another context.

### Strengths and limitations

This study explored the perspectives of those involved in managing and delivering multi-faceted support to prison leavers, highlighting how partnerships are integral to these types of interventions and the factors that influence this type of work. An established framework for conducting process evaluations was applied as a lens for data analysis, one which, to our knowledge, has had limited application in the field of offender rehabilitation. Challenges associated with recruiting participants from the community rehabilitation company and the local housing association in this study meant that the perspectives of individuals from these organizations, and as such any additional themes that may have been identified, were not captured in the findings. Another limitation associated with the sample is the study not collecting the experiences of prison leavers who took part in the pilot project. Due to limited resources, it was not feasible to interview prison leavers and so the study focused on exploring the perspectives of individuals managing and delivering the project. However, the inclusion of public advisers in the steering group ensured that the perspectives of people with lived experience of the criminal justice system were embedded within the design of the evaluation and the interpretation of the findings.

## Conclusion

Approaching the re-integration of prison leavers into the community through a combination of peer mentoring, sport and physical activity, and signposting, supported by close partnership working, was viewed positively by those involved in managing and delivering services in the pilot project. Many factors influencing the implementation of the project and partnership working identified from this evaluation correspond to those found in other areas of the wider literature and examples of similar interventions. The findings of this evaluation can be used to address the barriers identified (e.g., safeguarding concerns, clarification of offer, sustainability) and maintain what is working well (e.g., lived experience of peer mentors, information sharing, effective communication) to improve project delivery and partnership working in interventions aimed at supporting community re-integration for prison leavers. A larger-scale implementation and evaluation of the intervention, which triangulates both process and outcome measures, is needed to assess how the pilot project can be rolled out and scaled up in another context (e.g., geographical setting, population group).

## Supplementary Material

Appendix_1_COREQ_Checklist_Prison_Leaver_Project.docx

## Data Availability

The data generated and analyzed during this study are not publicly available as the details participants provided in the interviews/focus groups could lead to the identification of participants, but data are available from the corresponding author upon reasonable request.
